# Supplementary material for: Resveratrol Mitigates Metabolism in Human Microglia Cells
Source: Antioxidants (Basel). 2023 Jun 9;12(6):1248. doi: 10.3390/antiox12061248 (PMC10294838; doi:10.3390/antiox12061248)
Supplement: Supplementary file 1 [file antioxidants-12-01248-s001.zip › antioxidants-2442182-supplementary.pdf]

Supporting information for:

Resveratrol mitigates metabolism in human microglia cells

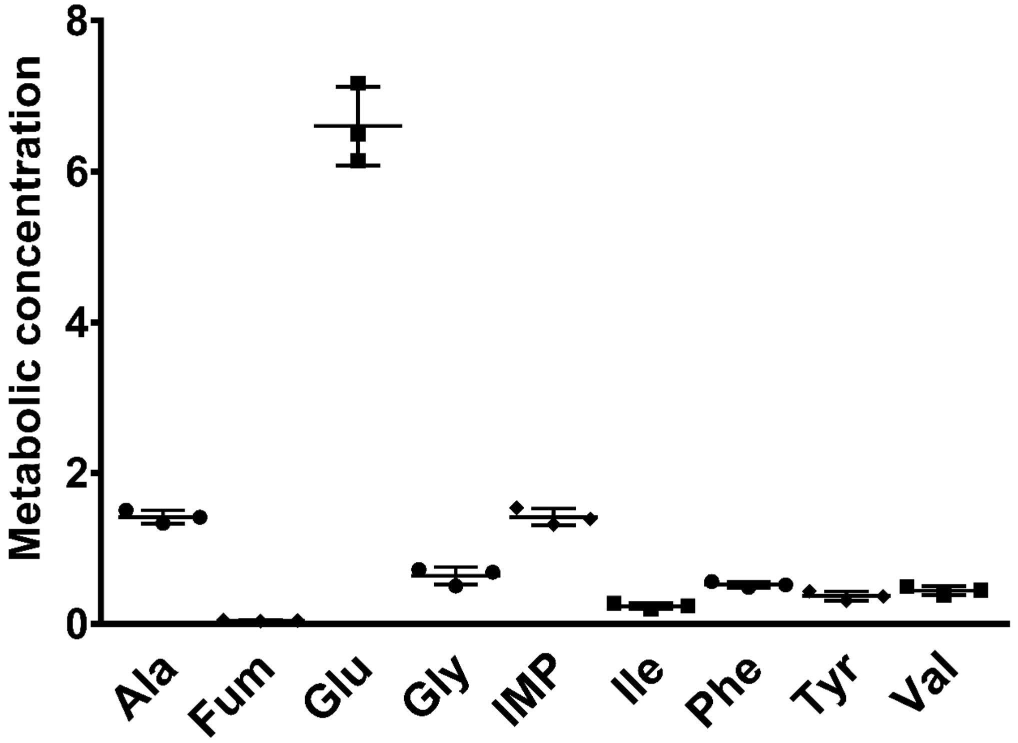

Figure S1. Reproducibility ^{1}H-NMR: Good reproducibility of ^{1}H-NMR sample preparation, measurement, and analysis. Individual results from three independent control measurements. HMC3 were co-treated with/without 100 µM resveratrol (RES) and compared to untreated control. Solvent = PEG400, n = 3 number of independent cell cultures.

Table S1. Resonance assignations of metabolites identified in ^{1}H NMR spectra of human HMC3 microglia cells.

Metabolite (Abbreviation)	^{1}H chemical shifts ppm (multiplicity)
Alanine (Ala)	1.45 (d)
Fumarate (Fum)	6.50 (s)
Glutamate (Glu)	2.35 (m)

Glycine (Gly)	3.54 (s)
Inosine monophosphate (IMP)	6.13 (d), 8.25 (s), 8.50 (s)
Isoleucine (Ile)	0.99 (s)
Phenylalanine (Phe)	7.27 (m), 7.45 (m)
Tyrosine (Tyr)	6.90 (d), 7.20 (d)
Valine (Val)	1.04 (d)
